# mTOR inhibition improves the immunomodulatory properties of human bone marrow mesenchymal stem cells by inducing COX-2 and PGE_2_

**DOI:** 10.1186/s13287-017-0744-6

**Published:** 2017-12-29

**Authors:** Binsheng Wang, Yu Lin, Yongxian Hu, Wei Shan, Senquan Liu, Yulin Xu, Hao Zhang, Shuyang Cai, Xiaohong Yu, Zhen Cai, He Huang

**Affiliations:** 1 Bone Marrow Transplantation Center, The First Affiliated Hospital, School of Medicine, Zhejiang University, Hangzhou, Zhejiang Province 310003 People’s Republic of China; 2 Institute of Hematology, Zhejiang University, Hangzhou, 310003 China

**Keywords:** Mesenchymal stem cells, Immunosuppressive properties, Immunogenicity, TSC-mTOR pathway, Rapamycin

## Abstract

**Background:**

Bone marrow mesenchymal stem cells (MSCs) are promising candidates for the treatment of various inflammatory disorders due to their profound immunomodulatory properties. However, the immunosuppressive capacity of MSCs needs activation by an inflammatory microenvironment, which may negatively impact the therapeutic effect because of increased immunogenicity. Here we explore the role of mammalian target of rapamycin (mTOR) signaling on the immunosuppressive capacity of MSCs, and its impact on immunogenicity in the inflammatory microenvironment.

**Methods:**

Human bone marrow MSCs were cocultured with activated human peripheral blood mononuclear cells, CD4^+^ T cells, and mouse splenocytes to evaluate the immunosuppressive function. Immunosuppressive factors were assessed by quantitative real-time polymerase chain reaction (PCR), Western blot, and enzyme-linked immunosorbent assay (ELISA). The expression of major histocompatibility complex (MHC) was detected by flow cytometry. Short hairpin (sh)RNA was used to downregulate tuberous sclerosis complex (TSC)2, TSC1, and cyclooxygenase (COX)-2 in MSCs.

**Results:**

Inhibition of mTOR signaling using rapamycin enhanced the immunosuppressive functions of MSCs, while prolonged exposure to rapamycin did not. The enhancement of the immunosuppressive function was independent of the inflammatory microenvironment, and occurred mainly through the upregulation of COX-2 and prostaglandin-E_2_ (PGE_2_) expression. Furthermore, mTOR inhibition did not impact the immunogenicity of MSCs. However, the upregulated expression of MHC class II molecules by interferon (IFN)-γ was attenuated by mTOR inhibition, whereas TSC2 knockdown had the opposite effect.

**Conclusions:**

These results reveal that the mTOR signaling pathway regulates MSC immunobiology, and short-term exposure to rapamycin could be a novel approach to improve the MSC-based therapeutic effect.

**Electronic supplementary material:**

The online version of this article (doi:10.1186/s13287-017-0744-6) contains supplementary material, which is available to authorized users.

## Background

Bone marrow mesenchymal stem/stromal cells (MSCs) are members of the hematopoietic stem cell (HSC) niche that have the capacity of hematopoiesis support and mesodermal lineage differentiation [1, 2]. Due to robust immunomodulatory and tissue reparative properties, MSCs have been widely applied to treat inflammatory conditions including graft-versus-host disease (GVHD) and autoimmune disease [3, 4]. Not only innate but also adaptive immune responses are modulated by MSCs. Numerous studies have shown that MSCs can shift macrophages phenotype, inhibit natural killer (NK) cell cytotoxicity, suppress T-cell activation, and promote the induction of regulatory T cells (Tregs) [5–8]. In addition to contact-dependent mechanisms, several soluble factors are involved in the immunosuppressive effects of MSCs, including transforming growth factor (TGF)-β1, interleukin (IL)-10, indoleamine 2,3-dioxygenase (IDO), prostaglandin-E_2_ (PGE_2_), and hepatocyte growth factor (HGF) [9–11]. However, the molecular mechanisms underlying the secretion of soluble factors remain to be elucidated.

Despite the favorable outcome of cell-based therapy in animal models and clinical settings, the immunosuppressive properties of MSCs are not constitutive but rather elicited by inflammatory cytokines such as interferon (IFN)-γ, tumor necrosis factor (TNF)-α, IL-1α, or IL-1β [12, 13]. Indeed, inflammatory cytokines, especially IFN-γ, upregulate the expression of immunosuppressive factors and activate the immunomodulatory function of MSCs [14, 15]. However, the major histocompatibility complex (MHC) molecule expression is also upregulated by inflammatory cytokines, in contrast to the low immunogenicity of resting MSCs [16–18]. Enhanced immunogenicity increases MSC lysis by T cells, and may negatively affect their allograft survival [19, 20]. Thus, strategies to induce the immunomodulatory capacity of MSCs whilst circumventing the enhancement of immunogenicity have gained much interest in recent years.

The mammalian target of rapamycin (mTOR) signaling plays a critical role in regulating cell growth, proliferation, and autophagy. mTOR complex 1 (mTORC1) and mTORC2 are two distinct complexes formed by mTOR and adapter proteins. The tuberous sclerosis complex (TSC)2 forms a heterodimer with TSC1, and negatively regulates mTORC1 activity [21]. The TSC-mTOR pathway is important in mediating inflammatory cytokine production of innate immune cells, as well as participating in the activation, differentiation, and function of adaptive immune cells [22, 23]. The mTOR inhibitor rapamycin has been reported to exert a synergistic effect when cotransplanted with MSCs to treat immune-related disease [24]. Moreover, rapamycin has a direct effect on the immunoregulatory potency of MSCs, although the mechanism is not fully elucidated [25, 26]. In addition, enhanced mTORC1 activity decreases MHC class II molecule expression in TSC1-deficient dendritic cells [27]. However, whether the immunomodulatory properties and MHC expression of MSCs are regulated by the TSC-mTOR pathway has not been clarified.

In this study, we sought to identify strategies to upregulate the immunomodulatory capacity of MSCs whilst circumventing the enhancement of their immunogenic property. We describe that mTOR inhibition in MSCs upregulated their immunosuppressive functions through a paracrine mechanism involving cyclooxygenase (COX)-2 and downstream PGE_2_. The immunogenicity was not enhanced by mTOR inhibition. Additionally, the enhancement of MHC-II expression by inflammatory cytokines was further attenuated by mTOR inhibition; knockdown of TSC2 showed the opposite effect. Our findings demonstrate mTOR inhibition as a promising approach to improving the therapeutic effect of MSCs in the treatment of immune-related diseases.

## Methods

### Cells and cell culture

Human bone marrow mononuclear cells were isolated from posterior iliac crest aspirates by density gradient centrifugation (Ficoll 1.077 g/ml; TBD) after each donor’s informed consent and approval from the ethics committee of The First Affiliated Hospital of Zhejiang University (reference number 2016-82). All experiments were performed in accordance with the approved guidelines. The cells were seeded into a T25 flask at 4 × 10^5^ cells/cm^2^ with low-glucose Dulbecco’s modified Eagle’s medium (DMEM; Gibco, Invitrogen) supplemented with 10% fetal bovine serum (FBS; Gibco), 100 U/ml penicillin, and 100 μg/ml streptomycin at 37 °C under a 5% CO_2_ atmosphere. The culture medium was changed every 3 days. When they reached ≥ 80% confluence, cells were subcultured at 8 × 10^3^ cells/cm^2^. MSCs from the third to the sixth passages were used for all experiments; these cells have been confirmed previously by measuring of a group of cell surface markers and by detecting multipotent differentiation potential [28].

Human peripheral blood mononuclear cells (PBMCs) were isolated from buffy coats of healthy volunteers by density gradient centrifugation. Human CD4 T cells were purified from PBMCs using CD4 T-cell enrichment cocktail (StemCell Technologies) according to the manufacturer’s protocols.

Splenocytes were isolated from C57BL/6 mouse spleen, and erythrocytes were removed with red blood cell lysis buffer (Biolegend). All animal experiments were approved by the animal ethics committee of The First Affiliated Hospital of Zhejiang University (reference number 2016-116) and carried out in accordance with the approved guidelines.

### Flow cytometry

Cells were stained with the fluorochrome-conjugated monocolonal antibodies against CD4-FITC (BD Biosciences, 555346), CD25-PE (Biolegend, 302606), CD127-APC (Biolegend, 351315), HLA-ABC-PE (Biolegend, 311406), HLA-DR-FITC (Biolegend, 307604), isotype control-FITC (eBioscience, 11-4714), isotype control-PE (eBioscience, 12-4714), and isotype control-APC (BD Biosciences, 550854). After washing with phosphate-buffered saline (PBS), the cells were subsequently detected by flow cytometry using FC500MCL (Beckman Coulter) and data were calculated using FlowJo Software (Tree Star).

### Immunosuppression assay

MSCs were plated in round-bottomed 96-well plates (0.1 × 10^4^ cells/well for 1:50, 0.125 × 10^4^ cells/well for 1:40, 0.25 × 10^4^ cells/well for 1:20, 0.5 × 10^4^ cells/well for 1:10, 1 × 10^4^ cells/well for 1:5, and 2 × 10^4^ cells/well for 1:2.5) and incubated at 37 °C overnight. Before coculturing with PBMCs, MSCs were pretreated with 1 nM, 10 nM, or 100 nM mTOR inhibitor (rapamycin; Selleck Chemicals) for 4 h, followed by washing with PBS three times to remove residual rapamycin. Freshly isolated PBMCs were labeled with 5 μM carboxyfluorescein diacetate succinimidyl ester (CFSE; Invitrogen) and stimulated with 2 μg/ml phytohemagglutinin (PHA; Sigma) and 100 U/ml IL-2 (Peprotech). PBMCs (5 × 10^4^) were cocultured with MSCs at different ratios in RPMI-1640 medium (Gibco) containing 10% FBS, 100 U/ml penicillin/streptomycin, and 50 μM β-mercaptoethanol (Gibco). In some experiments, PBMCs were cultured with 100 μl fresh RPMI-1640 medium and 100 μl out of 500 μl supernatant from 2 × 10^4^ or 4 × 10^4^ MSCs, which were cultured for 48 h after being pretreated with 10 nM or 100 nM rapamycin. Following a period of 5 days, PBMCs were harvested and analyzed. In some other experiments, COX-2 inhibitor (NS-398; Cayman Chemical) was added to the coculture system. For Treg induction assays, 2 × 10^4^ MSCs were cocultured with 2 × 10^5^ CD4 T cells in 24-well plates for 5 days. For detecting xenogeneic immunosuppression, MSCs were cocultured with 1 × 10^5^ mouse splenocytes for 5 days in the presence of 1 μg/ml anti-CD3/CD28 antibodies (BD Biosciences) and 100 U/ml IL-2, and then analyzed by flow cytometry.

### Enzyme-linked immunosorbent assay (ELISA)

MSCs were pretreated with 100 nM rapamycin for 4 h, followed by washing with PBS three times. Cells were then stimulated with or without 10 ng/ml TNF-α (Peprotech) plus 20 ng/ml IFN-γ (Peprotech) for 48 h. An ELISA kit (Cayman Chemical) was used to detect the PGE_2_ levels in the MSC culture supernatant according to the manufacturer’s instructions.

### shRNA knockdown

To knockdown TSC2, TSC1, or COX-2 of MSCs, short hairpin (sh)RNAs were constructed into the pGLV2-U6-Puro lentiviral vector (GenePharma). The targeting sequences were as follows: shTSC2_1, 5’-CGACGAGTCAAACAAGCCAAT-3’ [29]; shTSC2_2, 5’-CACTGGCCTTGGACGGTATTG-3’ [30]; shTSC1_1, 5’- GACACACAGAATAGCTATG-3’ [31]; shTSC1_2, 5’- GGGAGGTCAACGAGCTCTATT-3’ [31]; shCOX-2_1, 5’-GCAACACTTGAGTGGCTATCA-3’; shCOX-2_2, 5’-GGAACGTTGTGAATAACATTC-3’. A scrambled shRNA (shNC, 5’-TTCTCCGAACGTGTCACGT-3’) was used as the negative control. For virus production, shRNA constructs were cotransfected with psPAX2 and pMD2.G plasmids into 293 T cells using a calcium phosphate transfection kit (Biowit Technologies). MSCs were infected with lentivirus for 8–10 h and replaced with fresh growth medium. After 3 days of infection, knockdown efficiency of MSCs was confirmed before being used in the following experiments.

### Western blot analysis

After treatment with various cytokines and inhibitors, MSCs were washed with cold PBS and lysed with RIPA lysis buffer (Boster) plus protease inhibitor cocktail (Thermo) and phosphatase inhibitor tablet (Roche). Equal amounts of protein lysates were resolved on 10% SDS-PAGE and transferred onto nitrocellulose (NC) membrane (Pall), followed by blocking with 5% skimmed milk in TBST (150 mM NaCl, 0.1% Tween 20, 25 mM Tris-HCl, pH 7.6) for 2 h at room temperature. The membrane was incubated with the primary antibodies overnight and washed with TBST. After incubating with secondary IRDye 680 goat anti-mouse or IRDye 800 goat anti-rabbit antibodies (LI-COR Biosciences), the membrane was detected using the Odyssey Infrared Imaging System (LI-COR Biosciences). The following primary antibodies were used: rabbit COX-2 antibody (Cayman Chemical, 160107), rabbit mPGES-1 antibody (Cayman Chemical, 160140), rabbit S6K1 antibody (Cell Signaling Technology, 9202), rabbit phospho-S6K1 (Thr389) antibody (Cell Signaling Technology, 9234), rabbit 4E-BP1 antibody (Cell Signaling Technology, 9644), rabbit phospho-4E-BP1 (Thr37/46) antibody (Cell Signaling Technology, 2855), mouse Akt antibody (Cell Signaling Technology, 2920), rabbit phospho-Akt (Ser473) antibody (Cell Signaling Technology, 4060), rabbit phospho-GSK-3β (Ser9) antibody (Abcam, ab75814), and mouse β-actin antibody (Sigma, A5441).

### Quantitative real-time polymerase chain reaction (PCR)

Total RNA was prepared from MSCs using TRIzol reagent (Invitrogen) according to the suggested instructions. cDNA was synthesized from the RNA using the PrimeScript™ RT reagent Kit (Takara). Real-time quantitative PCR was performed using SYBR Premix Ex Taq™ (Takara) in a 10-μl reaction mixture containing specific primers (Additional file 1: Table S1). Each sample was run in triplicate, and expression was normalized to the endogenous reference (GAPDH). All the amplifications were performed on a LightCycler 480 system (Roche) and data were analyzed with LightCycler analysis software.

### Statistical analysis

All data are presented as mean ± SD and analyzed using SPSS 18.0 software. The results were compared using Student’s *t* test for two groups and analysis of variance (ANOVA) for multiple groups. *p* < 0.05 was considered statistically significant.

## Results

### The mTOR inhibitor enhances the immunosuppressive effects of human bone marrow MSCs

**Fig. 1:**
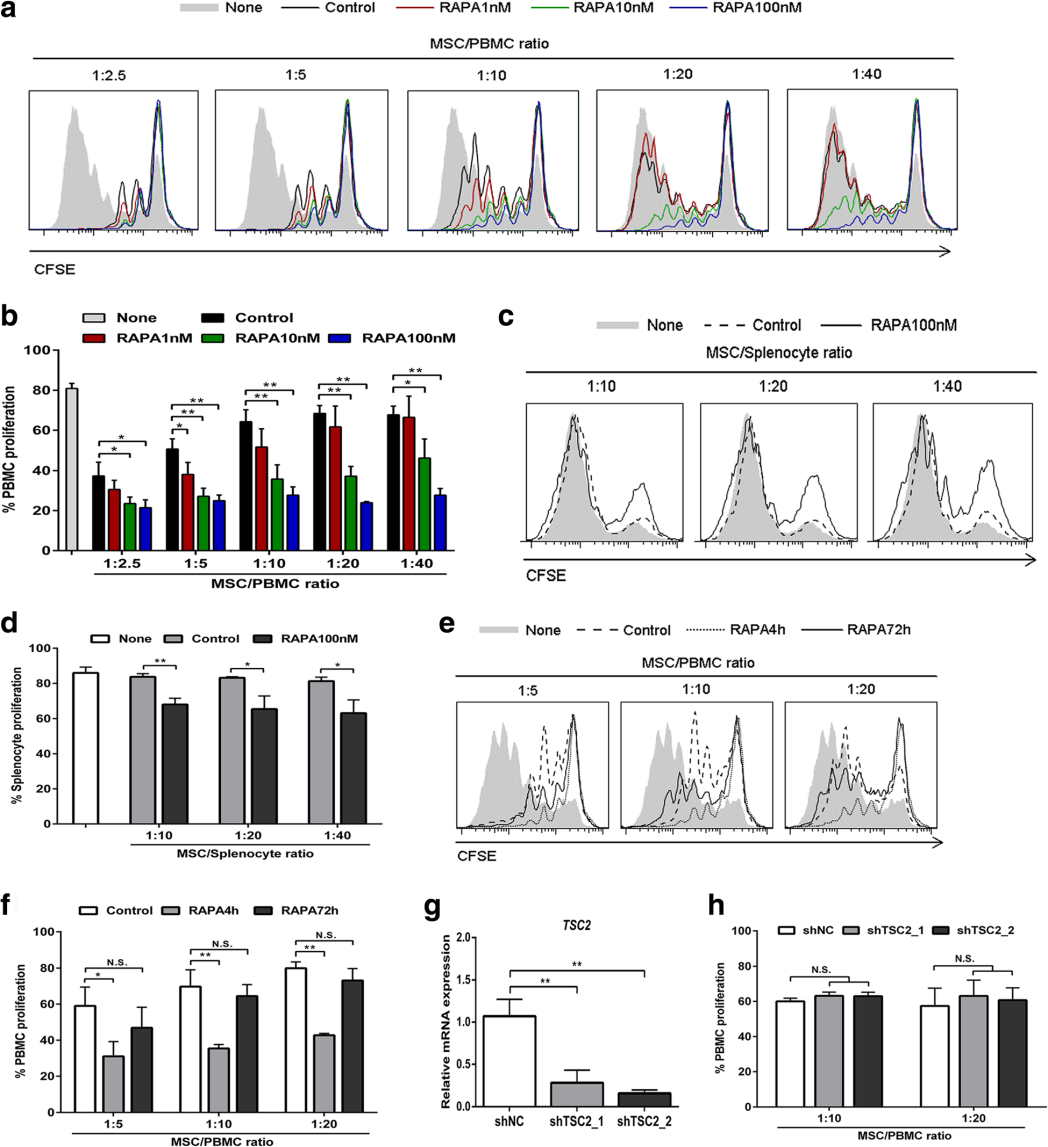
mTOR inhibition enhances the immunosuppressive functions of MSCs. **a**–**d** Mesenchymal stem cells (*MSCs*) were pretreated without or with rapamycin (*RAPA*) for 4 h, and cocultured with carboxyfluorescein diacetate succinimidyl ester (*CFSE*)-labeled human peripheral blood mononuclear cells (*PBMCs*) (**a**, representative data; **b**, pooled data) or mouse splenocytes (**c**, representative data; **d**, pooled data) for 5 days at the indicated ratio. PBMC and splenocyte proliferation were analyzed by flow cytometry. **e,f** MSCs were pretreated with 100 nM rapamycin for 4 h or 72 h, followed by coculturing with PBMCs for 5 days at the indicated ratio. PBMC proliferation was analyzed by flow cytometry (**e**, representative data; **f**, pooled data). **g** MSCs were infected with lentivirus carrying scrambled shRNA (shNC) or tuberous sclerosis complex (*TSC*)2-specific shRNAs (shTSC2_1, shTSC2_2). TSC2 knockdown efficiency was assessed by quantitative RT-PCR. **h** MSCs with or without TSC2 knockdown were cocultured with PBMCs at the indicated ratio. Flow cytometry showed the PBMC proliferation after 5 days. Data represent mean ± SD of at least three independent experiments. *p < 0.05, **p < 0.01. *N.S.* not significant

To determine whether TSC-mTOR signaling plays a functional role in the immunomodulatory properties of MSCs, we first inhibited mTOR activity using rapamycin. Human bone marrow MSCs were pretreated with various concentrations of rapamycin (1 nM, 10 nM, and 100 nM) for 4 h and cocultured with activated human PBMCs for 5 days. As revealed by the proliferation of PBMCs, pre-exposure to rapamycin significantly enhanced the immunosuppressive effects of MSCs (Fig. 1a and b; 1 nM: *p* < 0.05 only in 1:5 ratio; 10 nM: *p* < 0.05 in 1:2.5 and 1:40 ratio, *p* < 0.01 in 1:5, 1:10, and 1:20 ratio; 100 nM: *p* < 0.01 in all ratios except *p* < 0.05 in 1:2.5 ratio). We next examined whether mTOR inhibition could also modulate the xenogeneic immunoregulation properties of MSCs. Mouse splenocytes were activated with anti-CD3/CD28 antibodies and cocultured with MSCs. The proliferation of mouse splenocytes was not suppressed in the presence of MSCs (Fig. 1c and d), suggesting that the immunosuppressive functions of MSCs could not be elicited by mouse inflammatory cytokines. However, MSCs suppressed mouse splenocytes apparently when pretreated with rapamycin (Fig. 1c and d; *p* < 0.01 in 1:10 ratio, *p* < 0.05 in 1:20 and 1:40 ratio). MSCs have been documented with the capacity of inducing Tregs [8, 9]. However, our results showed that pre-exposure to rapamycin did not influence the proportion of Tregs induced by MSCs (Additional file 1: Figure S1), suggesting that Tregs may not underlie the enhanced immunosuppressive properties.

mTORC1 is highly sensitive to rapamycin but mTORC2 is relatively resistant, while prolonged treatment of rapamycin also inhibits mTORC2 activity [32]. Therefore, we prolonged the pretreatment of 100 nM rapamycin to 72 h to inhibit mTORC2 activity. In contrast to short-term pretreatment, prolonged pretreatment was unable to promote the immunosuppressive effects (Fig. 1e and f). To further investigate the role of TSC-mTOR signaling in regulating the immunomodulatory functions of MSCs, we silenced TSC2 expression using lentivirus carrying specific shRNAs. After confirming the efficiency of depletion (Fig. 1g; *p* < 0.01), MSCs were cocultured with PBMCs. However, knockdown of TSC2 showed no reduction in the immunosuppressive effects (Fig. 1h). The same results were obtained when TSC1 expression was knocked down (Additional file 1: Figure S2). Taken together, these results demonstrate that mTOR inhibition in MSCs enhances their immunomodulatory properties, and mTORC1 is mainly involved in this enhancement.

### The TSC-mTOR pathway regulates the expression of HLA molecules in human bone marrow MSCs

**Fig. 2:**
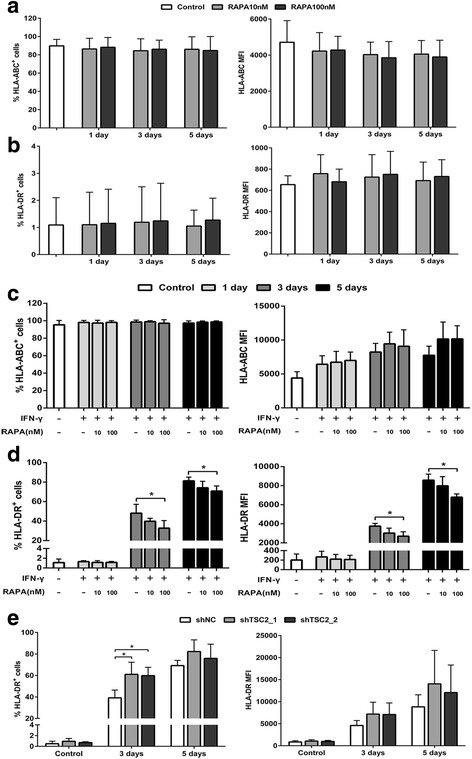
The TSC-mTOR pathway regulates the expression of HLA-DR but not HLA-ABC in IFN-γ-treated MSCs. Flow cytometry analysis of human leukocyte antigen (*HLA*)-ABC (**a**) or HLA-DR (**b**) positive cell proportion (*left*) and mean fluorescence intensity (*MFI*; *right*) at 1, 3, and 5 days after pretreatment with rapamycin (*RAPA*) for 4 h. Cells without pretreatment with rapamycin were indicated as control. **c,d** MSCs were pretreated with rapamycin followed by treatment with 10 ng/ml interferon gamma (*IFN-γ*) for 1, 3, and 5 days. The HLA-ABC (**c**) or HLA-DR (**d**) positive cell proportion (*left*) and MFI (*right*) were assessed by flow cytometry. Cells without treatment with rapamycin and IFN-γ were indicated as control. **e** MSCs without or with tuberous sclerosis complex (*TSC*)2 knockdown were treated with 10 ng/ml IFN-γ for 0, 3, and 5 days. HLA-DR positive cell proportion (*left*) and MFI (*right*) were assessed by flow cytometry. Data represent mean ± SD of at least four independent experiments. *p < 0.05. *shNC* lentivirus carrying scrambled shRNA

Human bone marrow MSCs express human leukocyte antigen (HLA) class I molecules but lack the expression of HLA class II and costimulatory molecules [2, 19]. We next investigated whether mTOR inhibition could alter the immunogenicity of MSCs whilst enhancing their immunosuppressive ability. We found that MSCs only constitutively expressed HLA-ABC, as indicated by previous studies, and that mTOR inhibition had no apparent influence on either HLA molecule expression (Fig. 2a and b), suggesting the absence of an immunogenic effect of mTOR inhibition. Since HLA molecule expression can be enhanced by the inflammatory microenvironment [16, 19], we examined whether mTOR inhibition is involved in HLA molecule expression in the presence of IFN-γ. As expected, IFN-γ enhanced the mean fluorescence intensity (MFI) of HLA-ABC despite the stable cell proportion; pretreatment with rapamycin had little further effect (Fig. 2c). Differentially, HLA-DR expression was significantly upregulated by IFN-γ including both cell proportion and MFI after 3 days. However, pre-exposure to 100 nM but not 10 nM rapamycin obviously attenuated the enhancement of HLA-DR expression by IFN-γ (Fig. 2d; *p* < 0.05 for both cell proportion and MFI). To further ascertain the role of the TSC-mTOR pathway in regulating HLA-DR expression, we silenced TSC2 expression in MSCs. Contrary to mTOR inhibition, TSC2 deficiency showed a further increase in HLA-DR expression after treatment with IFN-γ (Fig. 2e; *p* < 0.05 for cell proportion).

### Enhancement of immunosuppressive properties by mTOR inhibition is independent of the inflammatory microenvironment

**Fig. 3:**
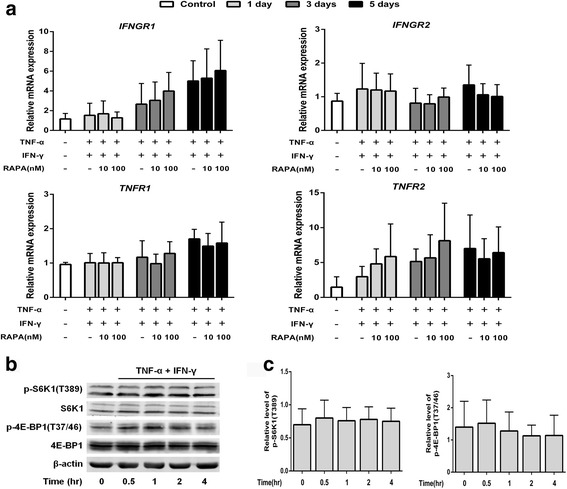
Enhancement of the immunosuppressive function by mTOR inhibition has no involvement with the inflammatory cytokine pathway. **a** MSCs were pretreated with 10 nM or 100 nM rapamycin (*RAPA*) followed by treatment with 10 ng/ml tumor necrosis factor alpha (*TNF-α*) plus 20 ng/ml interferon gamma (*IFN-γ*) for 1, 3, and 5 days. mRNA expression of the IFN-γ and TNF-α receptors *IFNGR1*, *IFNGR2*, *TNFR1*, and *TNFR2* was measured by quantitative RT-PCR. Cells without treatment with rapamycin and inflammatory cytokines were indicated as control. **b,c** MSCs were treated with 10 ng/ml TNF-α plus 20 ng/ml IFN-γ for the indicated time. The activation of mTOR substrates was determined by Western blot (**b**, representative data; **c**, pooled data). β-actin was used as internal control. Data represent mean ± SD of at least three independent experiments

Inflammatory cytokines elicit the immunomodulatory capacity when MSCs are exposed to the inflammatory microenvironment; thus we investigated whether the enhancement by mTOR inhibition was mediated by upregulation of the sensitivity of MSCs to inflammatory cytokines. The results from MSC coculturing with mouse splenocytes indicated that pretreatment with rapamycin could enhance the immunosuppressive functions without the activation by inflammatory cytokines (Fig. 1c and d). To further confirm this notion, we investigated the variation of receptors of inflammatory cytokines. Our results showed that pretreatment with rapamycin did not influence the expression of TNF-α and IFN-γ receptors (Additional file 1: Figure S3). When exposed to TNF-α plus IFN-γ, the expression of IFNGR1, TNFR1, and TNFR2 were upregulated after 5 days. However, pretreatment with rapamycin showed no further upregulation for all receptors (Fig. 3a). In addition, inflammatory cytokines had no effect on the mTOR signaling, as indicated by the phosphorylation of the substrates of mTOR (Fig. 3b and c). These results suggest that the enhancement of immunosuppressive functions by rapamycin is independent of the inflammatory microenvironment.

### Soluble factors are responsible for the enhancement in response to mTOR inhibition

**Fig. 4:**
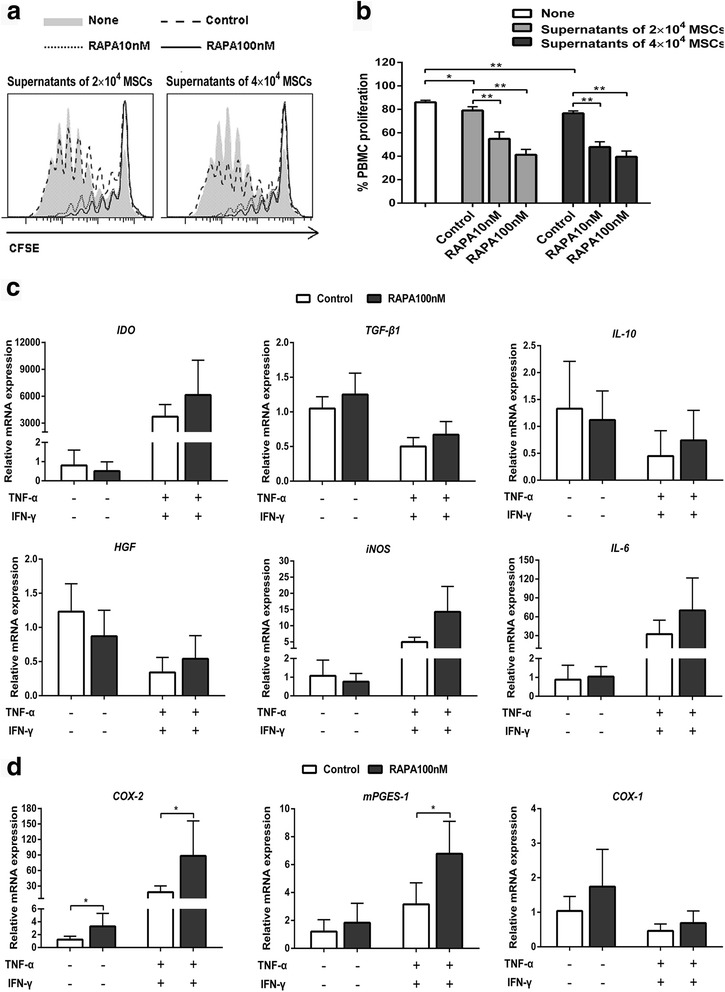
mTOR inhibition enhances the immunosuppressive properties through soluble factors. **a,b** Supernatants were collected after 48 h from rapamycin (*RAPA*)-pretreated mesenchymal stem cells (*MSCs*), followed by diluting 1:1 and adding peripheral blood mononuclear cells (*PBMCs*). PBMC proliferation was analyzed by flow cytometry (**a**, representative data; **b**, pooled data). **c** MSCs were pretreated with 100 nM rapamycin followed by treating with 10 ng/ml tumor necrosis factor alpha (*TNF-α*) plus 20 ng/ml interferon gamma (*IFN-γ*) for 24 h. The mRNA expression of immunosuppressive factors was measured by quantitative RT-PCR. **d** Quantitative RT-PCR analysis of *COX-2*, *mPGES-1*, and *COX-1* mRNA expression. Data represent mean ± SD of at least three independent experiments. *p < 0.05, **p < 0.01

Both cell-cell contact and soluble factors are involved in the immunosuppressive effects of MSCs [3, 4]; we therefore next explored which of these plays a fundamental role in the enhancement of the immunosuppressive effects by mTOR inhibition. We observed that in contrast to supernatants from normal cultured MSCs, supernatants from MSCs pretreated with rapamycin suppressed proliferation of PBMCs more significantly (Fig. 4a and b; *p* < 0.01 for both rapamycin concentrations). To investigate the underlying mechanisms, various immunosuppressive factors were detected. Pre-exposure to rapamycin did not influence the mRNA expression of *IDO*, *TGF-β1*, *IL-10*, *HGF*, *iNOS*, and *IL-6* regardless of the presence or not of TNF-α plus IFN-γ (Fig. 4c). Notably, MSCs pretreated with rapamycin expressed higher levels of *COX-2* mRNA (Fig. 4d; 2.64-fold, *p* < 0.05). Similarly, *COX-2* mRNA expression was remarkably elevated by pre-exposure to rapamycin when MSCs were subsequently treated with TNF-α plus IFN-γ (Fig. 4d; 4.86-fold, *p* < 0.05). mPGES-1, a PGE_2_ synthase, was also upregulated by mTOR inhibition in the presence of TNF-α plus IFN-γ (Fig. 4d; 2.15-fold, *p* < 0.05). The expression of another cyclooxygenase, COX-1, which is constitutively expressed in most tissues, was not affected by mTOR inhibition (Fig. 4d). These data suggest that soluble factors secreted by MSCs play the major role in promoting the effect of mTOR inhibition.

### mTOR inhibition enhances the immunosuppressive properties through increasing COX-2 and PGE_2_

**Fig. 5:**
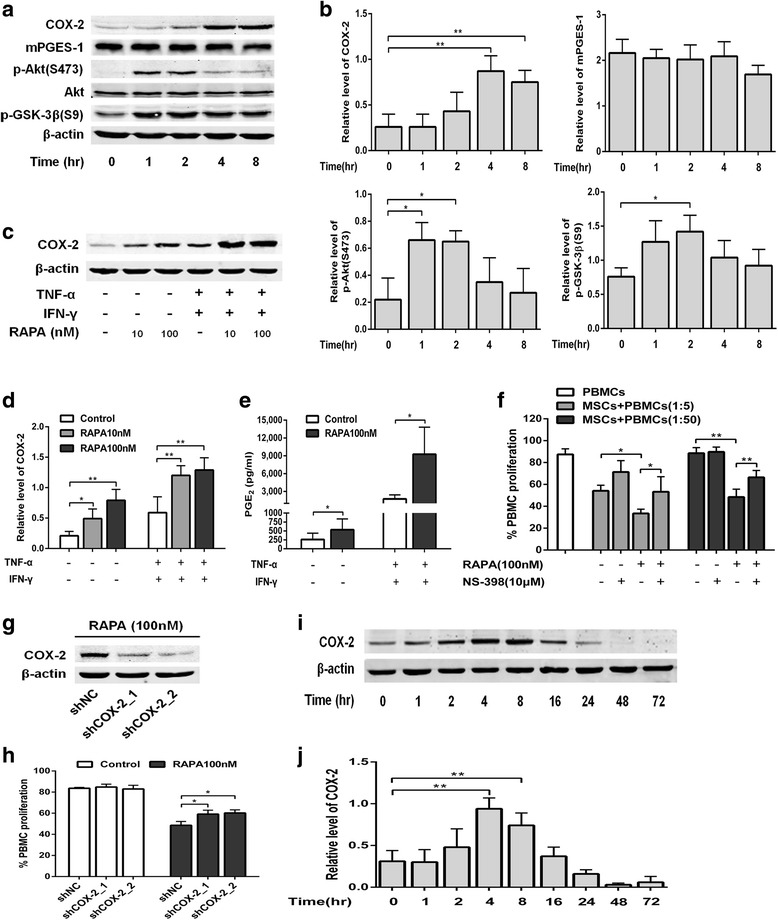
Critical roles of COX-2 and PGE_2_ in the enhancement of immunosuppressive properties induced by mTOR inhibition. **a,b** Mesenchymal stem cells (*MSCs*) were treated with 100 nM rapamycin (*RAPA*) for the indicated time. The activation and protein levels of COX-2, mPGES-1, Akt, GSK-3β were measured by Western blot (**a**, representative data; **b**, pooled data). **c,d** MSCs were pretreated with 10 nM or 100 nM rapamycin followed by treating with 10 ng/ml tumor necrosis factor alpha (*TNF-α*) plus 20 ng/ml interferon gamma (*IFN-γ*) for 8 h. The protein level of cyclooxygenase-2 (*COX-2*) was measured by Western blot (**c**, representative data; **d**, pooled data). **e** MSCs were pretreated with 100 nM rapamycin followed by treating with 10 ng/ml TNF-α plus 20 ng/ml IFN-γ for 48 h. Prostaglain-E_2_ (*PGE*_*2*_) in supernatants was measured by ELISA. **f** MSCs were pretreated with 100 nM rapamycin followed by coculturing with peripheral blood mononuclear cells (*PBMCs*) at the indicated ratio in the presence or not of 10 μM NS-398 (a COX-2-specific inhibitor). PBMC proliferation was analyzed by flow cytometry. **g** Western blot analysis of the COX-2 knockdown efficiency. **h** MSCs with or without COX-2 knockdown were pretreated with 100 nM rapamycin followed by coculturing with PBMCs at the ratio of 1:50. Flow cytometry showed the PBMC proliferation after 5 days. **i**,**j** MSCs were treated with 100 nM rapamycin for the indicated time. The protein level of COX-2 was measured by Western blot (**i**, representative data; **j**, pooled data). Data represent mean ± SD of at least three independent experiments. *p < 0.05, **p < 0.01. *shNC* lentivirus carrying scrambled short hairpin (sh)RNA

To further elucidate the role of COX-2 on the enhancement of immunosuppressive functions, we next evaluated COX-2 expression and activity after treatment with rapamycin. We found that rapamycin gradually increased the protein level of COX-2, while mPGES-1 was little affected (Fig. 5a and b; 3.36-fold for 4 h, *p* < 0.01; 2.88-fold for 8 h, *p* < 0.01). The phosphorylation of Akt at serine 473, which is associated with mTORC2 activity [21], was also upregulated by rapamycin. The phosphorylation of the Akt target GSK-3β was similarly upregulated (Fig. 5a and b). In addition, pre-exposure to rapamycin significantly increased the COX-2 expression in the presence of TNF-α plus IFN-γ (Fig. 5c and d; 2.05-fold for 10 nM rapamycin, *p* < 0.01; 2.20-fold for 100 nM rapamycin, *p* < 0.01). The production of downstream PGE_2_ in the supernatants of MSCs was increased consistently by pre-exposure to rapamycin whether in the presence of TNF-α plus IFN-γ (Fig. 5e; 5.28-fold, *p* < 0.05) or not (2.06-fold, *p* < 0.05).

We next used a COX-2-specific inhibitor, NS-398, to confirm the function of COX-2. The suppression of MSCs which was enhanced by mTOR inhibition was significantly abrogated in the presence of NS-398 (Fig. 5f; *p* < 0.05 in 1:5 ratio; *p* < 0.01 in 1:50 ratio), especially when MSCs were cocultured with PBMCs at the ratio of 1:50 (in which circumstance MSCs could not suppress the proliferation of PBMCs). When COX-2 expression was knocked down by shRNA (Fig. 5g), the proliferation of PBMCs was similarly modulated to the pattern of NS-398 (Fig. 5h; *p* < 0.05 for both shRNAs).

Since prolonged pretreatment with rapamycin was unable to promote the immunosuppressive effects of MSCs, we investigated whether COX-2 expression was affected by prolonged treatment with rapamycin. In contrast to short-term treatment, the protein level of COX-2 was gradually decreased after 8 h, even lower than the normal level after 48 h (Fig. 5i and j). It is noteworthy that when TSC2 was silenced the expression of COX-2 did not decline as expected (Additional file 1: Figure S4), which may explain the unaffected immunosuppressive effect after knockdown of TSC2. Taken together, these findings collectively support the idea that COX-2 and PGE_2_ are critical for the enhancement of immunosuppressive functions induced by mTOR inhibition.

## Discussion

MSCs have emerged as an attractive cell population for the treatment of GVHD and some autoimmune diseases due to their profound immunomodulatory properties [3, 4]. While the inflammatory microenvironment can elicit immunosuppressive activity, the immunogenicity of MSCs is also upregulated and negatively impacts the therapeutic effect in vivo [16–18]. Our results demonstrated that mTOR inhibition enhanced the immunosuppressive functions of MSCs mainly through upregulation of COX-2 and PGE_2_ expression. Moreover, mTOR inhibition reduced MHC-II expression in the inflammatory microenvironment. These findings suggest that the therapeutic potential of MSCs may be improved by manipulating the mTOR signaling pathway.

Protein kinase mTOR is involved in innate and adaptive immune responses of diverse cell types [22, 23]. mTOR interacts with different proteins to form mTORC1 or mTORC2. These have different downstream substrates as well as sensitivities to rapamycin [21]. mTORC1 and mTORC2 play differential roles in some cellular events, such as osteogenic and adipogenic differentiation of MSCs [33]. Our data showed that short-term pre-exposure to the mTOR inhibitor rapamycin enhanced the immunosuppressive functions of MSCs. However, when mTORC2 activity was disrupted by prolonged pre-exposure to rapamycin, the promoting effect disappeared, suggesting the differential roles of mTORC1 and mTORC2 in regulating the immunomodulatory properties of MSCs. Consistent with this, short-term treatment with rapamycin increased the expression of COX-2, while the expression gradually decreased with prolonged treatment. In addition, short-term treatment with rapamycin upregulated the phosphorylation of Akt at serine 473, suggesting a promoting effect on activation of mTORC2. Indeed, it has been reported that the mTORC1 substrate S6K1 reduces mTORC2 signaling by phosphorylating rictor, which is a core component of mTORC2 [34, 35]. Thus, a more detailed investigation with respect to regulation by mTORC1 and mTORC2 on the immunomodulatory properties of MSCs needs to be undertaken.

Previous studies have shown that the immunomodulatory capacity of MSCs is activated by inflammatory cytokines [12, 13]. However, the immunosuppression was not observed when MSCs were stimulated with mouse inflammatory cytokines. The enhancement of immunosuppression by pretreatment with rapamycin in this context indicated that the promoting effect of mTOR inhibition may be independent of inflammatory cytokine signaling. This was further confirmed by the unchanged expression of inflammatory cytokine receptors when pretreated with rapamycin.

PGE_2_ is a product catalyzed by cyclooxygenases from arachidonic acid [36]. PGE_2_ secreted from MSCs inhibits activated T-cell proliferation, NK cell cytotoxicity, and dendritic cell maturation [6, 37, 38]. We found that the enhancement of immunosuppressive functions by mTOR inhibition was dependent on soluble factors; PGE_2_ is the main immunosuppressive factor in supernatant. A recent study showed that brief incubations with immunosuppressive drugs, such as rapamycin, FK506, increased the ability to inhibit T-cell proliferation of MSCs as well as fibroblasts [25]. This cell type-independent potentiating effect of rapamycin was considered to be mediated by the adsorption of rapamycin and subsequent diffusion toward T cells. In contrast, our results do not support the idea because of the incapability of potentiating the immunosuppressive effects by prolonged treatment with rapamycin. Moreover, we demonstrated the fundamental role of PGE_2_ in the enhancement of immunosuppressive functions which was partially abrogated by NS-398 or knockdown of COX-2. Another study showed that pretreatment with rapamycin in adipose tissue-derived MSCs increased the capacity of suppressing Th17 cell expansion and upregulated the expression of IL-10, IDO, and TGF-β [26]. Nevertheless, rapamycin was incubated for 48 h at 50 nM in their study. The upregulation of the above immunosuppressive factors was not observed by pre-exposure to rapamycin regardless of the presence of inflammatory cytokines in our study; the discrepancies may be caused by different sources of MSCs and differences in incubation time and concentration of rapamycin.

The low immunogenicity of MSCs was considered as a positive for therapy in vivo. However, the upregulation of MHC expression by inflammatory cytokines increases the immunogenicity of MSCs, and thus limits their use [16, 20]. We found that, unlike inflammatory cytokines, mTOR inhibition in MSCs did not affect the expression of MHC molecules whilst it enhanced their immunosuppressive capacity. Moreover, the upregulation of MHC-II expression by IFN-γ was attenuated by pretreatment with rapamycin. As MHC expression is associated with recognition and lysis by T cells, whether mTOR inhibition enhanced the immunosuppressive capacity of MSCs through downregulating MHC expression which may be increased in coculture should be taken into consideration. First, in our study mTOR inhibition rapidly increased COX-2 and PGE_2_ expression, whereas the downregulation of MHC-II expression by mTOR inhibition occurred slowly. In addition, pretreatment with 10 nM rapamycin was potent in enhancing the immunosuppressive capacity of MSCs, whereas MHC-II expression was not affected at this concentration. Hence, the downregulation of MHC expression in coculture may not be the underlying mechanism of enhancement of immunosuppressive capacity by mTOR inhibition.

## Conclusions

**Fig. 6:**
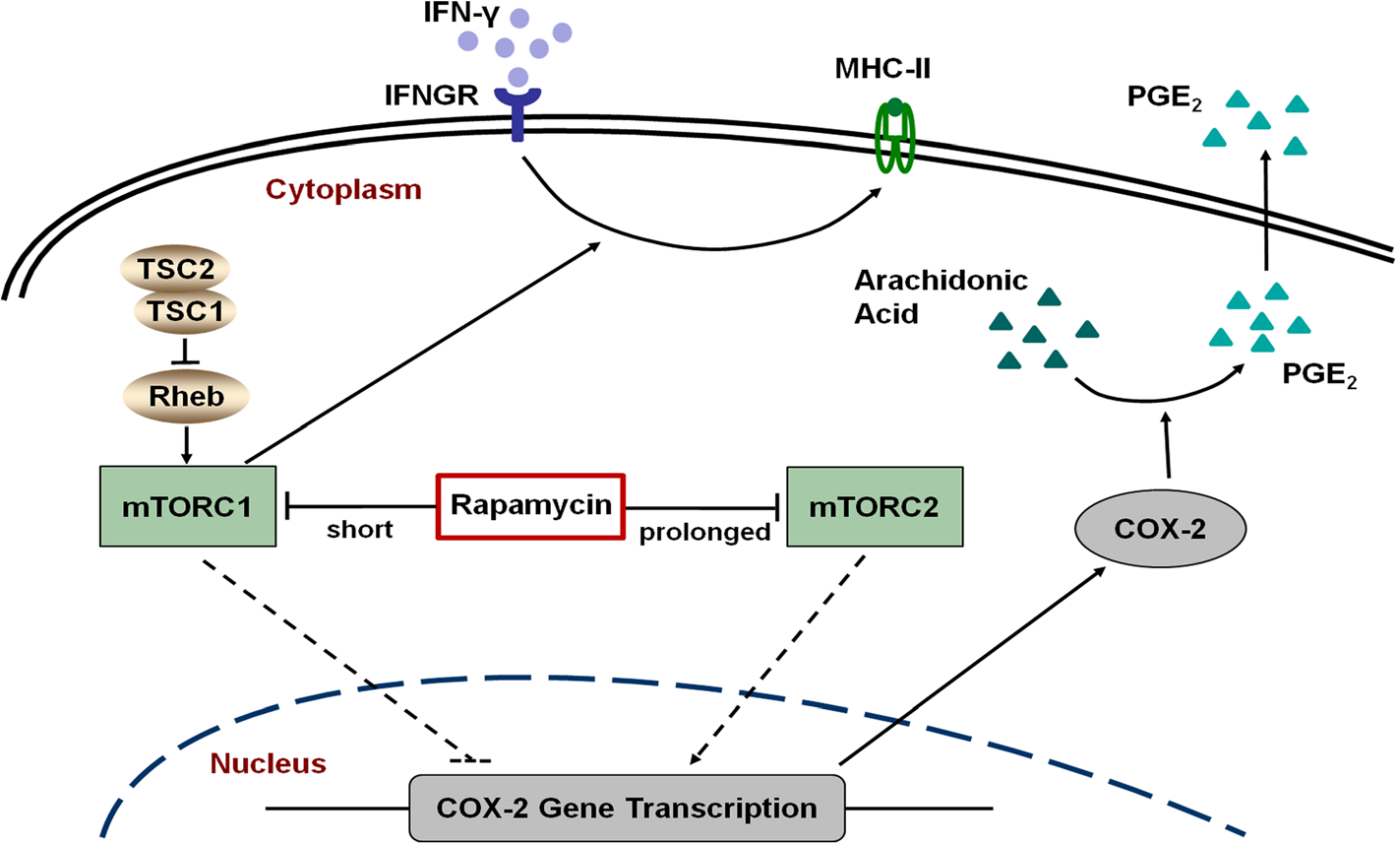
Schematic representation of the effect of the TSC-mTOR pathway on the immunosuppressive properties and immunogenicity of MSCs. Short-term exposure to rapamycin inhibits mTORC1 and promotes the transcription of COX-2 followed by increasing the secretion of PGE_2_, whereas prolonged exposure to rapamycin inhibits mTORC2 and decreases the expression of COX-2. Further investigations are needed to ascertain whether mTORC2 mediates the upregulation of COX-2 by mTORC1 inhibition. Short-term exposure to rapamycin also decreases the membrane expression of MHC-II, which is upregulated by IFN-γ; knockdown of TSC2 has an opposite effect. *COX-2* cyclooxygenase-2, *IFN* interferon, *IFNGR* interferon receptor, *MHC* major histocompatibility complex, *mTORC* mammalian target of rapamycin complex, *PGE*_*2*_ prostaglandin-E_2_, *TSC* tuberous sclerosis complex

In summary, the present study demonstrates that mTOR inhibition is involved in the immunosuppressive properties and immunogenicity of MSCs. While short-term pretreatment with rapamycin enhances the ability to suppress T-cell proliferation through COX-2 and PGE_2_, prolonged pretreatment with rapamycin has no such effect. In addition, short-term pretreatment with rapamycin attenuates the upregulation of MHC-II expression by IFN-γ (Fig. 6). Our results suggest that short-term incubation with rapamycin may represent a novel therapeutic strategy of MSCs for immunological disorders.

## Additional file

Additional file 1: Figure S1. mTOR inhibition has no effect on induction regulatory T cells of MSCs. MSCs were pretreated with or without rapamycin for 4 h and cocultured with human CD4 T cells for 5 days; the proportion of CD4^+^CD25^hi^CD127^–^ cells was analyzed by flow cytometry (A, representative data; B, pooled data). Data represent mean ± SD of four independent experiments. ***p* < 0.01. *N.S.* not significant. **Figure S2.** Knockdown of TSC1 has no effect on immunomodulatory functions of MSCs. (A) MSCs were infected with lentivirus carrying scrambled shRNA (shNC) or TSC1-specific shRNAs (shTSC1_1, shTSC1_2); TSC1 knockdown efficiency was assessed by quantitative RT-PCR. (B) MSCs with or without TSC1 knockdown were cocultured with PBMCs at the indicated ratio; flow cytometry showed the PBMC proliferation after 5 days. Data represent mean ± SD of three independent experiments. ***p* < 0.01. *N.S.* not significant. **Figure S3.** mTOR inhibition in MSCs does not influence the expression of inflammatory cytokine receptors. MSCs were pretreated with 10 nM or 100 nM rapamycin for 4 h; IFN-γ and TNF-α receptor *IFNGR1*, *IFNGR2*, *TNFR1*, *TNFR2* mRNA expression was measured by quantitative RT-PCR at the indicated time. Cells without pretreatment with rapamycin were indicated as control. Data represent mean ± SD of three independent experiments. **Figure S4.** Knockdown of TSC2 does not show an obvious difference in expression of COX-2. (A) MSCs with or without TSC2 knockdown were treated with 10 ng/ml TNF-α plus 20 ng/ml IFN-γ for 24 h. *COX-2* mRNA expression was measured by quantitative RT-PCR. (B) MSCs with or without TSC2 knockdown were treated with 10 ng/ml TNF-α plus 20 ng/ml IFN-γ for 8 h. The protein level of COX-2 was measured by Western blot. Data represent mean ± SD of three independent experiments. *N.S.* not significant. **Table S1.** Primer sequences used for real-time PCR. (ZIP 13342 kb)

## Abbreviations

CFSE: Carboxyfluorescein diacetate succinimidyl ester
COX: Cyclooxygenase
DMEM: Dulbecco’s modified Eagle’s medium
ELISA: Enzyme-linked immunosorbent assay
FBS: Fetal bovine serum
GVHD: Graft-versus-host disease
HGF: Hepatocyte growth factor
HLA: Human leukocyte antigen
HSC: Hematopoietic stem cell
IDO: Indoleamine 2,3-dioxygenase
IFN: Interferon
IL: Interleukin
MFI: Mean fluorescence intensity
MHC: Major histocompatibility complex
MSC: Mesenchymal stem cell
mTOR: Mammalian target of rapamycin
mTORC: mTOR complex
NK: Natural killer
PBMC: Peripheral blood mononuclear cell
PBS: Phosphate-buffered saline
PCR: Polymerase chain reaction
PGE_2_: Prostaglandin-E_2_
PHA: Phytohemagglutinin
sh: Short hairpin
TGF: Transforming growth factor
TNF: Tumor necrosis factor
Treg: Regulatory T cell
TSC: Tuberous sclerosis complex
