# Supplementary material for: mTOR inhibition improves the immunomodulatory properties of human bone marrow mesenchymal stem cells by inducing COX-2 and PGE2
Source: Stem Cell Res Ther. 2017 Dec 29;8:292. doi: 10.1186/s13287-017-0744-6 (PMC5747167; doi:10.1186/s13287-017-0744-6)
Supplement: Additional file 1: Figure S1. — mTOR inhibition has no effect on induction regulatory T cells of MSCs. MSCs were pretreated with or without rapamycin for 4 h and cocultured with human CD4 T cells for 5 days; the proportion of CD4+CD25hiCD127– cells was analyzed by flow cytometry (A, representative data; B, pooled data). Data represent mean ± SD of four independent experiments. **p < 0.01. N.S. not significant. Figure S2. Knockdown of TSC1 has no effect on immunomodulatory functions of MSCs. (A) MSCs were infected with lentivirus carrying scrambled shRNA (shNC) or TSC1-specific shRNAs (shTSC1_1, shTSC1_2); TSC1 knockdown efficiency was assessed by quantitative RT-PCR. (B) MSCs with or without TSC1 knockdown were cocultured with PBMCs at the indicated ratio; flow cytometry showed the PBMC proliferation after 5 days. Data represent mean ± SD of three independent experiments. **p < 0.01. N.S. not significant. Figure S3. mTOR inhibition in MSCs does not influence the expression of inflammatory cytokine receptors. MSCs were pretreated with 10 nM or 100 nM rapamycin for 4 h; IFN-γ and TNF-α receptor IFNGR1, IFNGR2, TNFR1, TNFR2 mRNA expression was measured by quantitative RT-PCR at the indicated time. Cells without pretreatment with rapamycin were indicated as control. Data represent mean ± SD of three independent experiments. Figure S4. Knockdown of TSC2 does not show an obvious difference in expression of COX-2. (A) MSCs with or without TSC2 knockdown were treated with 10 ng/ml TNF-α plus 20 ng/ml IFN-γ for 24 h. COX-2 mRNA expression was measured by quantitative RT-PCR. (B) MSCs with or without TSC2 knockdown were treated with 10 ng/ml TNF-α plus 20 ng/ml IFN-γ for 8 h. The protein level of COX-2 was measured by Western blot. Data represent mean ± SD of three independent experiments. N.S. not significant. Table S1. Primer sequences used for real-time PCR. (ZIP 13342 kb) [file 13287_2017_744_MOESM1_ESM.zip › Additional file 1.pdf]

mTOR inhibition improves the immunomodulatory properties of human bone marrow mesenchymal stem cells by inducing COX-2 and PGE_2

Binsheng Wang[1,2+], Yu Lin[1,2+], Yongxian Hu[1,2+], Wei Shan[1,2], Senquan Liu[1,2], Yulin Xu[1,2], Hao Zhang[1,2], Shuyang Cai[1,2], Xiaohong Yu[1,2], Zhen Cai[1,2], and He Huang[1,2*]

[1]Bone Marrow Transplantation Center, The First Affiliated Hospital, School of Medicine, Zhejiang University, Hangzhou, 310003, China

[2]Institute of Hematology, Zhejiang University, Hangzhou, 310003, China

[*]Corresponding author: He Huang

[+]These authors contributed equally to this work

Mail address: 79 Qingchun Road, Hangzhou, Zhejiang Province, PR China, 310003.

Tel: +86-571-8723-6706.   Fax: +86-571-8723-6562.

E-mail address: huanghe@zju.edu.cn

Supplementary figure legends

Supplementary Figure S1. mTOR inhibition has no effect on induction regulatory T cells of MSC. MSCs were pre-treated without or with rapamycin for 4 hr, and co-cultured with human CD4 T cells for 5 days, the proportion of $CD4^{+}CD25^{hi}CD127^{-}$ cells was analyzed by flow cytometry (A, representative data; B, pooled data). RAPA, rapamycin. Data represent mean ± SD of four independent experiments. **p < 0.01, N.S., not significant.

Supplementary Figure S2. Knockdown of TSC1 has no effect on immunomodulatory functions of MSC. (A) MSCs were infected with lentivirus carrying scrambled shRNA (shNC) or TSC1-specific shRNAs (shTSC1_1, shTSC1_2), TSC1 knockdown efficiency was assessed by quantitative RT-PCR. (B) MSCs without or with TSC1 knockdown were co-cultured with PBMCs at the indicated ratio, flow cytometry showed the PBMC proliferation after 5 days. Data represent mean ± SD of three independent experiments. **p < 0.01, N.S., not significant.

Supplementary Figure S3. mTOR inhibition in MSCs does not influence the expression of inflammatory cytokine receptors. MSCs were pre-treated with 10 nM or 100 nM rapamycin for 4 hr, IFN-γ and TNF-α receptors *IFNGR1*, *IFNGR2*, *TNFR1*, *TNFR2* mRNA expression were measured by quantitative RT-PCR at the indicated time. Cells without pre-treatment with rapamycin were indicated as control. RAPA, rapamycin. Data represent mean ± SD of three independent experiments.

Supplementary Figure S4. Knockdown of TSC2 does not show an obvious difference in expression of COX-2. (A) MSCs without or with TSC2 knockdown were treated with 10 ng/ml TNF-α plus 20 ng/ml IFN-γ for 24 hr. *COX-2* mRNA expression was measured by quantitative RT-PCR. (B) MSCs without or with TSC2 knockdown were treated with 10 ng/ml TNF-α plus 20 ng/ml IFN-γ for 8 hr. The protein level of COX-2 was measured by western blot. Data represent mean ± SD of three independent experiments. N.S., not significant.

Supplementary table

Table S1. Primer sequences used for real-time PCR.

Name	Primer sequences (5’-3’)
TSC2	F：TCACAGACAATGGGAGACACA<br>R：CAAGTTCACCAGCACCAGAA
TSC1	F：AGAGCGAGAGCCAGTGTGAT<br>R：GTGAGGGCCATCTAGGTTCA
COX-1	F：TACTGGAAGCCGAGCACATT<br>R：AGGGACAGGTCTTGGTGTTG
COX-2	F：TGAGTGTGGGATTTGACCAG<br>R：TGTGTTTGGAGTGGGTTTCA
mPGES-1	F：GGAACTTTCTGGTCCCTTCAG<br>R：TCTCAGGTCACGGGTCTAGG
IDO	F：TCACAGACCACAAGTCACAGC<br>R：TTGGCAGTAAGGAACAGCAAT
TGF-β1	F：TTGATGTCACCGGAGTTGTG<br>R：TGCAGTGTGTTATCCCTGCT
IL-10	F：CCACGCTTTCTAGCTGTTGA<br>R：GCTCCCTGGTTTCTCTTCCT
iNOS	F：CCTGGCAATGGAGAGAAACT<br>R：AGAACCGAGGGTACATGCTG

HGF	F：GCACTGACTCCGAACAGGAT<br>R：AGGAGGAGATGCAGGAGGAC
IL-6	F：CAAATTCGGTACATCCTCGAC<br>R：GCCATCTTTGGAAGGTTCAG
IFNGR1	F：ATGGAGACGAGCAGGAAGTC<br>R：TCGCTAACTGGCACTGAATCT
IFNGR2	F：CTCCTCTCCCTTTGACATCG<br>R：AAGCAGTTGTGCCTGGACTT
TNFR1	F：TCTCAACCCTCAACTGTCACC<br>R：CAGCTATGGCCTCTCACTCC
TNFR2	F：CAACTCCAGAACCCAGCACT<br>R：CACACCCACAATCAGTCCAA
GAPDH	F：AGAAGGCTGGGGCTCATTTG<br>R：AGGGGCCATCCACAGTCTTC
