# Supplementary figures and images for: mTOR inhibition improves the immunomodulatory properties of human bone marrow mesenchymal stem cells by inducing COX-2 and PGE2
Source: Stem Cell Res Ther. 2017 Dec 29;8:292. doi: 10.1186/s13287-017-0744-6 (PMC5747167; doi:10.1186/s13287-017-0744-6)

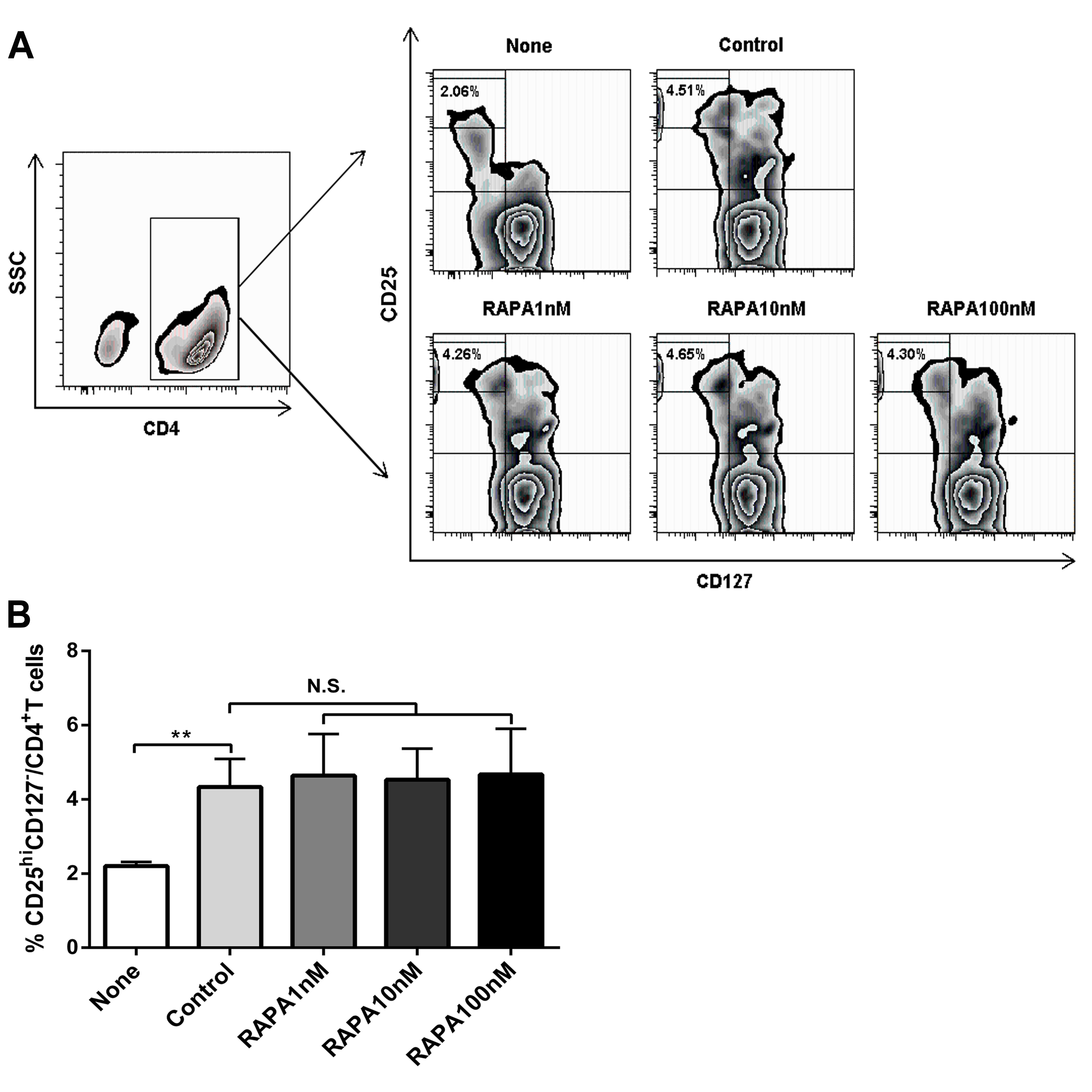

Supplement: Additional file 1: Figure S1. — mTOR inhibition has no effect on induction regulatory T cells of MSCs. MSCs were pretreated with or without rapamycin for 4 h and cocultured with human CD4 T cells for 5 days; the proportion of CD4+CD25hiCD127– cells was analyzed by flow cytometry (A, representative data; B, pooled data). Data represent mean ± SD of four independent experiments. **p < 0.01. N.S. not significant. Figure S2. Knockdown of TSC1 has no effect on immunomodulatory functions of MSCs. (A) MSCs were infected with lentivirus carrying scrambled shRNA (shNC) or TSC1-specific shRNAs (shTSC1_1, shTSC1_2); TSC1 knockdown efficiency was assessed by quantitative RT-PCR. (B) MSCs with or without TSC1 knockdown were cocultured with PBMCs at the indicated ratio; flow cytometry showed the PBMC proliferation after 5 days. Data represent mean ± SD of three independent experiments. **p < 0.01. N.S. not significant. Figure S3. mTOR inhibition in MSCs does not influence the expression of inflammatory cytokine receptors. MSCs were pretreated with 10 nM or 100 nM rapamycin for 4 h; IFN-γ and TNF-α receptor IFNGR1, IFNGR2, TNFR1, TNFR2 mRNA expression was measured by quantitative RT-PCR at the indicated time. Cells without pretreatment with rapamycin were indicated as control. Data represent mean ± SD of three independent experiments. Figure S4. Knockdown of TSC2 does not show an obvious difference in expression of COX-2. (A) MSCs with or without TSC2 knockdown were treated with 10 ng/ml TNF-α plus 20 ng/ml IFN-γ for 24 h. COX-2 mRNA expression was measured by quantitative RT-PCR. (B) MSCs with or without TSC2 knockdown were treated with 10 ng/ml TNF-α plus 20 ng/ml IFN-γ for 8 h. The protein level of COX-2 was measured by Western blot. Data represent mean ± SD of three independent experiments. N.S. not significant. Table S1. Primer sequences used for real-time PCR. (ZIP 13342 kb) [file 13287_2017_744_MOESM1_ESM.zip › Figure S1.tif]

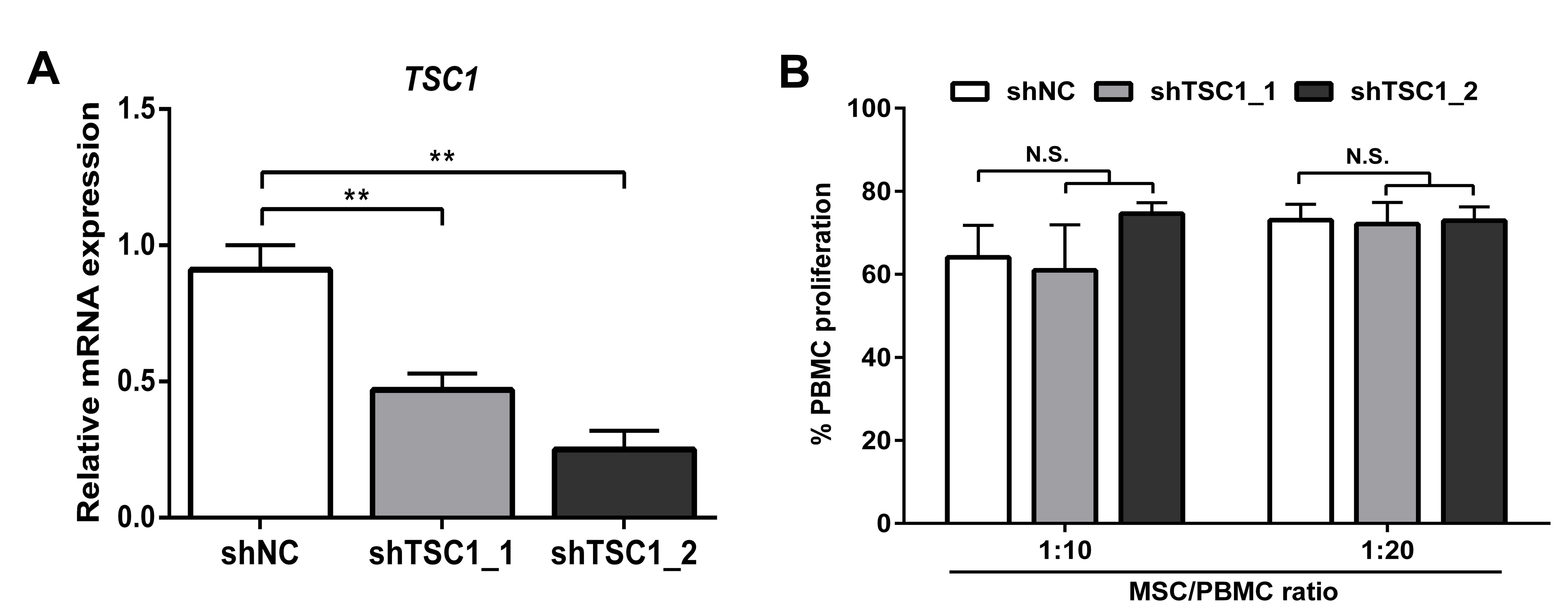

Supplement: Additional file 1: Figure S1. — mTOR inhibition has no effect on induction regulatory T cells of MSCs. MSCs were pretreated with or without rapamycin for 4 h and cocultured with human CD4 T cells for 5 days; the proportion of CD4+CD25hiCD127– cells was analyzed by flow cytometry (A, representative data; B, pooled data). Data represent mean ± SD of four independent experiments. **p < 0.01. N.S. not significant. Figure S2. Knockdown of TSC1 has no effect on immunomodulatory functions of MSCs. (A) MSCs were infected with lentivirus carrying scrambled shRNA (shNC) or TSC1-specific shRNAs (shTSC1_1, shTSC1_2); TSC1 knockdown efficiency was assessed by quantitative RT-PCR. (B) MSCs with or without TSC1 knockdown were cocultured with PBMCs at the indicated ratio; flow cytometry showed the PBMC proliferation after 5 days. Data represent mean ± SD of three independent experiments. **p < 0.01. N.S. not significant. Figure S3. mTOR inhibition in MSCs does not influence the expression of inflammatory cytokine receptors. MSCs were pretreated with 10 nM or 100 nM rapamycin for 4 h; IFN-γ and TNF-α receptor IFNGR1, IFNGR2, TNFR1, TNFR2 mRNA expression was measured by quantitative RT-PCR at the indicated time. Cells without pretreatment with rapamycin were indicated as control. Data represent mean ± SD of three independent experiments. Figure S4. Knockdown of TSC2 does not show an obvious difference in expression of COX-2. (A) MSCs with or without TSC2 knockdown were treated with 10 ng/ml TNF-α plus 20 ng/ml IFN-γ for 24 h. COX-2 mRNA expression was measured by quantitative RT-PCR. (B) MSCs with or without TSC2 knockdown were treated with 10 ng/ml TNF-α plus 20 ng/ml IFN-γ for 8 h. The protein level of COX-2 was measured by Western blot. Data represent mean ± SD of three independent experiments. N.S. not significant. Table S1. Primer sequences used for real-time PCR. (ZIP 13342 kb) [file 13287_2017_744_MOESM1_ESM.zip › Figure S2.tif]

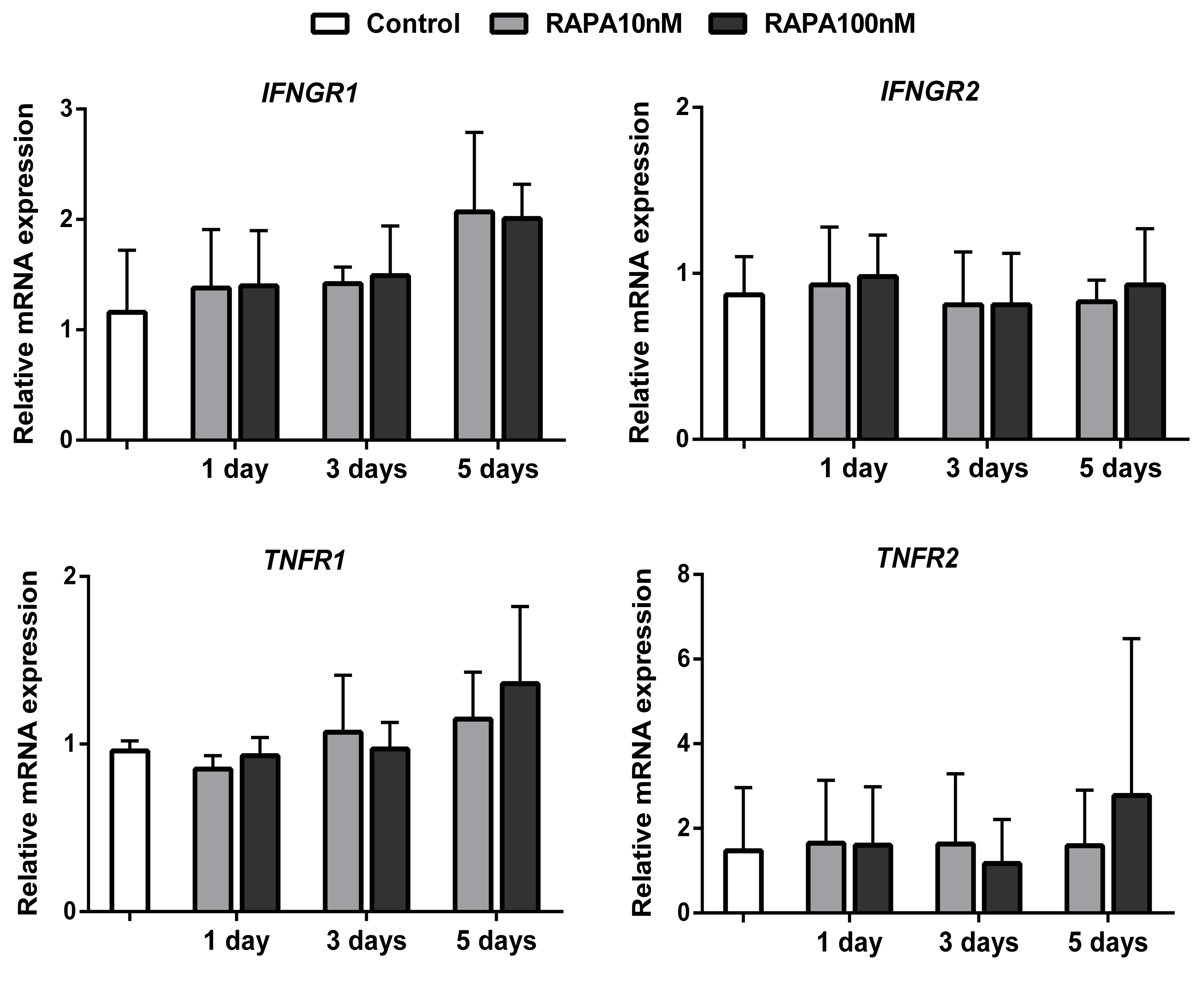

Supplement: Additional file 1: Figure S1. — mTOR inhibition has no effect on induction regulatory T cells of MSCs. MSCs were pretreated with or without rapamycin for 4 h and cocultured with human CD4 T cells for 5 days; the proportion of CD4+CD25hiCD127– cells was analyzed by flow cytometry (A, representative data; B, pooled data). Data represent mean ± SD of four independent experiments. **p < 0.01. N.S. not significant. Figure S2. Knockdown of TSC1 has no effect on immunomodulatory functions of MSCs. (A) MSCs were infected with lentivirus carrying scrambled shRNA (shNC) or TSC1-specific shRNAs (shTSC1_1, shTSC1_2); TSC1 knockdown efficiency was assessed by quantitative RT-PCR. (B) MSCs with or without TSC1 knockdown were cocultured with PBMCs at the indicated ratio; flow cytometry showed the PBMC proliferation after 5 days. Data represent mean ± SD of three independent experiments. **p < 0.01. N.S. not significant. Figure S3. mTOR inhibition in MSCs does not influence the expression of inflammatory cytokine receptors. MSCs were pretreated with 10 nM or 100 nM rapamycin for 4 h; IFN-γ and TNF-α receptor IFNGR1, IFNGR2, TNFR1, TNFR2 mRNA expression was measured by quantitative RT-PCR at the indicated time. Cells without pretreatment with rapamycin were indicated as control. Data represent mean ± SD of three independent experiments. Figure S4. Knockdown of TSC2 does not show an obvious difference in expression of COX-2. (A) MSCs with or without TSC2 knockdown were treated with 10 ng/ml TNF-α plus 20 ng/ml IFN-γ for 24 h. COX-2 mRNA expression was measured by quantitative RT-PCR. (B) MSCs with or without TSC2 knockdown were treated with 10 ng/ml TNF-α plus 20 ng/ml IFN-γ for 8 h. The protein level of COX-2 was measured by Western blot. Data represent mean ± SD of three independent experiments. N.S. not significant. Table S1. Primer sequences used for real-time PCR. (ZIP 13342 kb) [file 13287_2017_744_MOESM1_ESM.zip › Figure S3.tif]

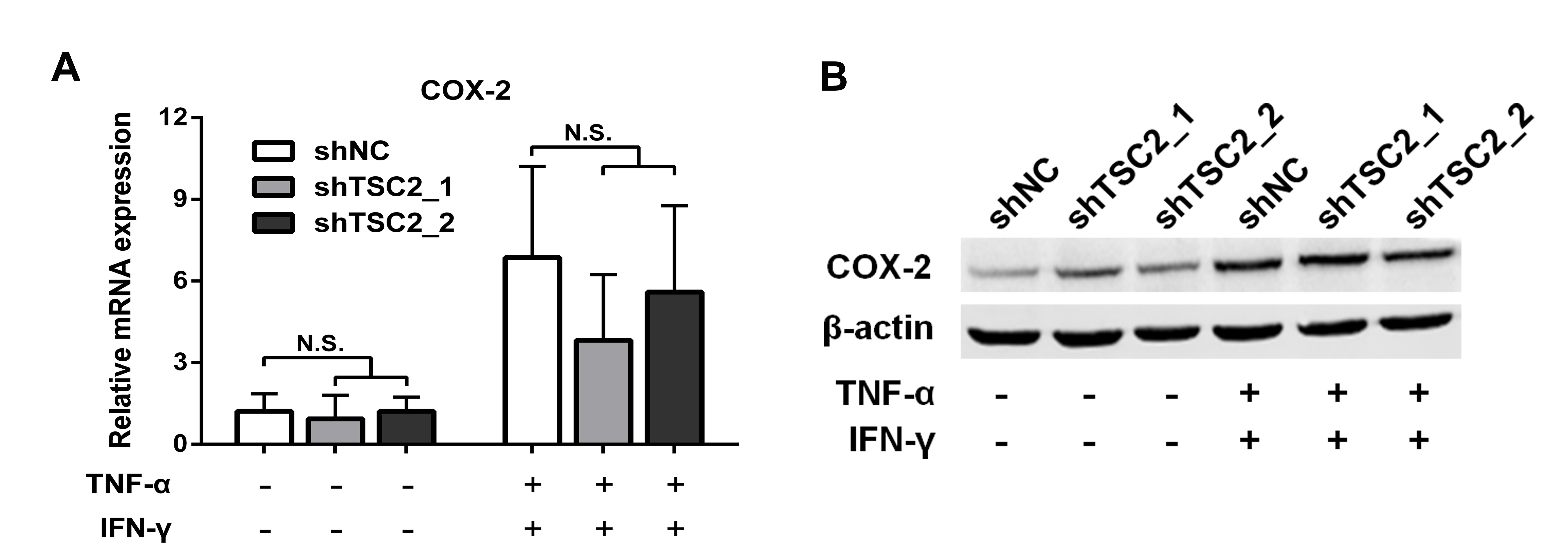

Supplement: Additional file 1: Figure S1. — mTOR inhibition has no effect on induction regulatory T cells of MSCs. MSCs were pretreated with or without rapamycin for 4 h and cocultured with human CD4 T cells for 5 days; the proportion of CD4+CD25hiCD127– cells was analyzed by flow cytometry (A, representative data; B, pooled data). Data represent mean ± SD of four independent experiments. **p < 0.01. N.S. not significant. Figure S2. Knockdown of TSC1 has no effect on immunomodulatory functions of MSCs. (A) MSCs were infected with lentivirus carrying scrambled shRNA (shNC) or TSC1-specific shRNAs (shTSC1_1, shTSC1_2); TSC1 knockdown efficiency was assessed by quantitative RT-PCR. (B) MSCs with or without TSC1 knockdown were cocultured with PBMCs at the indicated ratio; flow cytometry showed the PBMC proliferation after 5 days. Data represent mean ± SD of three independent experiments. **p < 0.01. N.S. not significant. Figure S3. mTOR inhibition in MSCs does not influence the expression of inflammatory cytokine receptors. MSCs were pretreated with 10 nM or 100 nM rapamycin for 4 h; IFN-γ and TNF-α receptor IFNGR1, IFNGR2, TNFR1, TNFR2 mRNA expression was measured by quantitative RT-PCR at the indicated time. Cells without pretreatment with rapamycin were indicated as control. Data represent mean ± SD of three independent experiments. Figure S4. Knockdown of TSC2 does not show an obvious difference in expression of COX-2. (A) MSCs with or without TSC2 knockdown were treated with 10 ng/ml TNF-α plus 20 ng/ml IFN-γ for 24 h. COX-2 mRNA expression was measured by quantitative RT-PCR. (B) MSCs with or without TSC2 knockdown were treated with 10 ng/ml TNF-α plus 20 ng/ml IFN-γ for 8 h. The protein level of COX-2 was measured by Western blot. Data represent mean ± SD of three independent experiments. N.S. not significant. Table S1. Primer sequences used for real-time PCR. (ZIP 13342 kb) [file 13287_2017_744_MOESM1_ESM.zip › Figure S4.tif]
